# Economic evaluations of immunization programs as an indispensable tool for policymakers

**DOI:** 10.1186/s12913-023-10071-z

**Published:** 2023-11-22

**Authors:** Saskia den Boon, Sayem Ahmed, Abdur Razzaque Sarker

**Affiliations:** 1 World Health Organization, Geneva, Switzerland; 2 Health Economics and Health Technology Assessment, School of Health and Wellbeing, University of Glasgow, Glasgow, UK; 3 Health Economics and Financing Research, Bangladesh Institute of Development Studies, Dhaka, Bangladesh; 4 Health Economics Unit, Centre for Health Policy, The University of Melbourne, Melbourne, Australia

## Abstract

Introducing new vaccines within national immunization programs requires careful consideration of disease- and vaccine-related issues as well as of the strength of the program and the affected health system. Economic evaluations play an essential role in this process. In this editorial, we set the context and invite contributions for a BMC Health Services Research Collection of articles titled ‘Economic Evaluations of Vaccine Programs’.

## Main text

Vaccination is one of the most successful public health interventions in history, with great impact on burden and mortality associated with infectious diseases. Immunization also positively impacts populations beyond the immunized individuals by reducing transmission of infections and through herd immunity. The World Health Organization (WHO) estimates that immunization saves 3.5–5 million lives each year [1]. In addition to health benefits, vaccination programs bring economic benefits; they contribute to reducing healthcare expenditure, by preventing the overload of the health care system, averting productivity losses, and diminishing health inequalities among the population [2]. Furthermore, immunization is positively related to future school enrolment and cognitive development of adolescents [3, 4]. A recent study also observed that immunization may reduce households’ catastrophic financial burden [5].

When the WHO established the Expanded Programme on Immunization (EPI, now the Essential Programme on Immunization) in 1974, it included vaccines against six childhood vaccine-preventable diseases (diphtheria, pertussis, tetanus, poliomyelitis, measles, and tuberculosis). Since then, biomedical advances and new technologies led to the development of new vaccines for both existing and emerging diseases, including for example, the recent use of new mRNA technology for the development of effective COVID-19 vaccines. As a result, WHO now has recommendations for routine vaccinations against 23 diseases, including those for the protection of older children, adolescents, and adults.

The decision to introduce a new, safe, effective, and authorized vaccine into national vaccination programs relies on policymakers and is based on a multitude of factors and a comprehensive analysis of the available evidence. Disease burden analysis and modelling estimates of the impact of the vaccine on health outcomes e.g. cases or deaths averted as well as quality-adjusted life year (QALY) gained, are essential to this aim. In addition to health burden estimates, programmatic decision-making can also greatly benefit from information on the economic burden that the vaccine-preventable disease poses on society. In this context, a cost-of-illness study estimates the direct and indirect cost resulting from a given vaccine-preventable disease. However, evaluating whether the costs of purchasing and delivering a new vaccine are justified by its preventive benefits, requires economic evaluations in which costs *and* health outcomes of alternative strategies are compared. Cost-effectiveness analysis (CEA) and cost-utility analysis (CUA) determine the incremental cost-effectiveness ratios (ICER), calculated as the difference in cost between two possible interventions, divided by the difference in their effect measured in natural units, QALYs or disability-adjusted life years (DALYs). This standardization allows for comparing the new intervention with alternative interventions or competing health priorities. As such, these analyses can support decision-makers in choosing between interventions and allocating resources.

There are several methodological considerations when conducting economic evaluations [6]. The model should cover a sufficiently long time horizon to capture the long-term benefits of vaccine programs. Further, the economic model should take into account the population-wide impact, including herd immunity, rather than only the individual-level impact. Increasingly, it is recognized that economic evaluations should capture the wider impact of vaccines, such as health equity or the impact on the wider economy. Extended cost-effectiveness analysis typically addresses health gains, financial risk protection benefits, total cost to the policymaker and the distribution of effects [7] while Full Value of Vaccine Assessments (FVVA) are proposed to capture broader benefits of vaccines as well as opportunity costs borne by stakeholders. The deliberate process of FVVA ensures equitable vaccine access and coverage, and sustainable impact [8]. Another consideration for governments and funders and for the applicable modeling approach is that new vaccines cannot be evaluated in isolation and should be evaluated as part of a package of interventions. In this regard, constrained optimization modelling allows for the determination of the optimal mix of prevention interventions for an infectious disease subject to budget and feasibility constraints [9].

Further, against a background of scarce resources and competing priorities, Budget Impact Analysis (BIA) can capture the all-possible cost of adding a new vaccine to a routine immunization program and observe the effect of this addition on the health budget over time. Indeed, financial sustainability is a critical issue in resource-poor settings, since new vaccines are relatively expensive and their purchase often relies on global donor commitment [10, 11]. For countries with higher incomes, long-term financial sustainability and mobilization of sufficient domestic resources are particularly important to guarantee. In these contexts, innovative financing models might be required [12]. Fiscal impact modeling could complement cost-effectiveness analysis to consider the broader consequences for governments attributed to vaccines. Fiscal modeling evaluates how investments in immunization programs influence government public accounts and tax revenue and give an estimate of the Return on Investment (ROI) or Net Present Value (NPV) of vaccination programs [13].

People in Low- or Middle-Income Countries (LMICs) are more vulnerable to the transmission of vaccine-preventable diseases [14]. However, there is a dearth of evidence on costs associated with immunization programs and evaluation of their effectiveness in these settings. For instance, a systematic review of the cost-effectiveness of the rotavirus vaccine conducted in 2019 showed that among 102 selected studies, only one third were from LMICs [15]. In these settings, economic analyses of vaccination programs essential to support investment decisions by governments and development partners. Contributions from LMICs to this Collection are, therefore, strongly encouraged.

In consideration of the complexity of the available tools and of the diverse scenarios for their application, this collection welcomes a range of articles on the different types of economic evaluations outlined above as well as those on vaccine delivery costs, methodological approaches of economic evaluation related to vaccines, predictive models of vaccine programs, and vaccine policy assessment. This collection on economic analyses of vaccination programs will be useful to guide health policymakers, and benefit researchers and academicians to move forward with innovative research in this area.

## Data Availability

Not applicable.
